# Adaptation to Extreme Environments in an Admixed Human Population from the Atacama Desert

**DOI:** 10.1093/gbe/evz172

**Published:** 2019-08-06

**Authors:** Lucas Vicuña, Mario I Fernandez, Cecilia Vial, Patricio Valdebenito, Eduardo Chaparro, Karena Espinoza, Annemarie Ziegler, Alberto Bustamante, Susana Eyheramendy

**Affiliations:** 1 Department of Statistics, Faculty of Mathematics, Pontificia Universidad Católica de Chile, Santiago, Chile; 2 Department of Urology, Clínica Alemana, Santiago, Chile; 3 Center for Genetics and Genomics, Faculty of Medicine, Clínica Alemana Universidad del Desarrollo, Santiago, Chile; 4 Department of Urology, Hospital Regional, Antofagasta, Chile; 5 Faculty of Engineering and Sciences, Universidad Adolfo Ibañez, Peñalolén, Santiago, Chile

**Keywords:** arsenic, positive selection, bladder cancer, Native Americans

## Abstract

Inorganic arsenic (As) is a toxic xenobiotic and carcinogen associated with severe health conditions. The urban population from the Atacama Desert in northern Chile was exposed to extremely high As levels (up to 600 µg/l) in drinking water between 1958 and 1971, leading to increased incidence of urinary bladder cancer (BC), skin cancer, kidney cancer, and coronary thrombosis decades later. Besides, the Andean Native-American ancestors of the Atacama population were previously exposed for millennia to elevated As levels in water (∼120 µg/l) for at least 5,000 years, suggesting adaptation to this selective pressure. Here, we performed two genome-wide selection tests—PBS_*n*__1_ and an ancestry-enrichment test—in an admixed population from Atacama, to identify adaptation signatures to As exposure acquired before and after admixture with Europeans, respectively. The top second variant selected by PBS_*n*__1_ was associated with *LCE4A-C1orf68*, a gene that may be involved in the immune barrier of the epithelium during BC. We performed association tests between the top PBS_*n*__1_ hits and BC occurrence in our population. The strongest association (*P *=* *0.012) was achieved by the *LCE4A-C1orf68* variant. The ancestry-enrichment test detected highly significant signals (*P *=* *1.3 × 10^−9^) mapping *MAK16*, a gene with important roles in ribosome biogenesis during the G1 phase of the cell cycle. Our results contribute to a better understanding of the genetic factors involved in adaptation to the pathophysiological consequences of As exposure.

## Introduction

Inorganic arsenic (As) is a toxic xenobiotic and carcinogen associated with the occurrence of severe health conditions, including increased mortality in early life, cardiovascular and liver toxicity, and cancer (Schlebusch et al. 2015). One of the cancers with the higher risk due to chronic As exposure is urinary bladder cancer (BC) (Chen et al. 2005; Chung et al. 2013; Saint-Jacques et al. 2018). Several factors seem to contribute to As-induced carcinogenesis, including epigenetic alterations, impairment of DNA-repair and oxidative stress (Huang et al. 2004; Chung et al. 2013; Sinha et al. 2013). Also, As induces cell-cycle arrest by suppressing cell-cycle checkpoint proteins (Flora 2011). Although some epigenetic factors (i.e., methylation of genes) have been significantly associated with As-induced BC (Chen et al. 2007; Yang et al. 2014), the contribution of genetic polymorphisms to this condition is less understood. One study found that a locus in the As-detoxification pathway enzyme GSTM1 is significantly associated with an increased BC risk in an As-exposed cohort from Taiwan. This observation suggests individual genetic susceptibility to BC related to As exposure (Chung et al. 2013).

**Fig. 1.**
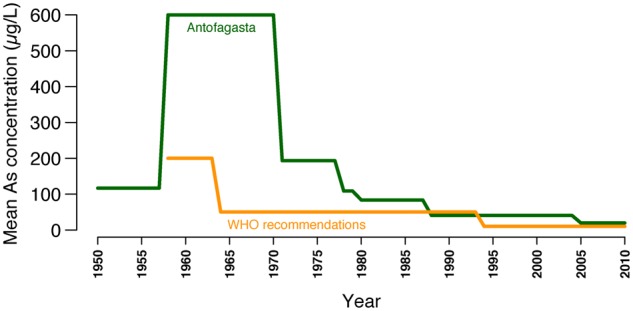
—Mean As concentrations in water in Antofagasta between 1950 and 2010. These measurements were compared with the WHO recommendations for As concentration in drinking water. Adapted from Fernandez et al. (2012), Elsevier, @ Copyright 2012.

In nature, As is produced by volcanic activity and is usually deposited in nearby rivers and lakes. In Andean regions, some rivers show high As levels in water. One of them is the Loa River, the only water supplier of the Antofagasta region of northern Chile, located in the hyperarid Atacama Desert. The Loa and its tributaries have As concentrations of 100–1,000 µg/l (Romero et al. 2003). Several native Andean populations have lived across the Loa basin for at least 12,000 years (Latorre et al. 2013; Falabella et al. 2017). This suggests an early human consumption of high As levels in water. In fact, 5,000–7,000-year-old mummies showing high As levels in their bodies have been found in the Atacama Desert (Byrne et al. 2010; Yáñez et al. 2015). Arguably, such populations adapted to this selective pressure, leaving genetic adaptation signatures in their genomes and in the genomes of their modern admixed descendants. Indeed, adaptive genetic loci in the key As-metabolizing enzyme AS3MT were found in populations with high Native-American ancestry from northern Chile (Apata et al. 2017) and Argentina (Eichstaedt et al. 2015; Schlebusch et al. 2015) historically exposed to As. However, it is unknown which are the organ and tissue systems over which genes such as *AS3MT* are acting to increase evolutionary fitness. In 1958, As concentrations in drinking water in Antofagasta—the largest city of the Atacama Desert—increased from 117 to 600 µg/l after the incorporation of two As-rich rivers as water sources. Thereafter, this population was exposed to significantly elevated As levels until 1971, when the first water treatment plant began its operations. Afterward, the installation of cleaner water sources led to As concentrations <10 µg/l during the last decade (Smith et al. 2018) (fig. 1). As a consequence, BC incidence and BC-specific mortality rates increased dramatically in this population since the 1970s and have remained high during the last years (Fernandez et al. 2012). In fact, BC incidence in this region was 4.1 times higher among men (20.6 vs. 5.0/100,000) and 4.3 times higher among women (8.1 vs. 1.9/100,000) than that of a comparable region within Chile during 2008–2010 (Espinoza et al. 2017; Galaz et al. 2017). However, these differences are significantly smaller than those observed in other populations with similar levels of As exposure (>100 µg/l), such as Taiwan. Here, BC incidence in affected regions was 7.9 times higher among men (26.06 vs. 3.31/100,000) and 18.0 times higher among women (21.10 vs. 1.17/100,000) when compared with that of the rest of the country in 1981–1985 (Chiang et al. 1993). There are no further studies comparing BC incidence rates between exposed and nonexposed populations, in part because few populations worldwide have been exposed to high As levels in water (Christoforidou et al. 2013).

BC incidence in South America is the lowest in males and the second lowest in females when compared across ten world regions (supplementary fig. 1, Supplementary Material online) (Ferlay and Ervik 2018). Because BC heritability is estimated to be ∼31% (Lichtenstein et al. 2000) and most South American countries have high Native-American genetic ancestry proportions, Native-American genetic variants may in part underlie this lower BC incidence.

In the present study, we searched for Native-American genetic signatures of adaptation to As exposure in admixed people from Atacama, by performing three approaches. First, we implemented the PBS*n*1 test of positive selection. PBS*n*1 is a normalized version of Population Branch Statistic (PBS) (Crawford et al. 2017), which has strong power to detect recent selective sweeps acting mostly on standing variation (Yi et al. 2010). We used this test to detect adaptive loci in local people acquired between the split time of their ancestors from Mesoamericans ∼12,000 years ago (ya) (Harris et al. 2018) and before admixture with Europeans. Second, we performed associations between putative loci selected by PBS*n*1 and the occurrence of BC in the Atacama cohort. This approach is suitable to elucidate function when sample sizes are small (Ilardo et al. 2018) and has proven successful in identifying loci underlying important physiological adaptations in Tibetans (Yi et al. 2010), Inuit (Fumagalli et al. 2015), and “Sea Nomads” of Southeast Asia (Ilardo et al. 2018). Third, we implemented an ancestry-enrichment test (Bryc et al. 2010; Bhatia et al. 2014), which captures a different kind of positive selection; namely, admixture-facilitated adaptation acting through gene flow (Jeong et al. 2014). We used this test to detect adaptation in local people acquired after admixture with Europeans.

By properly correcting for admixture, PBS*n*1 identified putative selected variants acquired by the Native-American ancestors of the Atacama population. The top second locus was associated with *LCE4A-C1orf68*, a gene involved in the barrier function of the epithelium that may play a role in immune defense during BC. The strongest association between loci selected by PBS*n*1 and BC was achieved by the aforementioned *LCE4A-C1orf68* variant (*P *=* *0.012). The ancestry-enrichment test detected several significant single-nucleotide polymorphisms (SNPs) in the whole cohort. Among them, some were also significantly enriched for Native-American ancestry in controls but not in cases, suggesting that postadmixture adaptation to As had an impact on the urinary bladder. Among those, the most significant variants (*P *=* *1.3 × 10^−9^) mapped the genes *MAK16* and *FUT10*, which are in high linkage disequilibrium (LD) with *TTI2*. *MAK16* encodes a ribosomal protein with an important role in ribosome biogenesis during the cell cycle (Wickner 1988; Kater et al. 2017), whereas *FUT10* and *TTI2* are also related to the cell cycle.

In summary, our results suggest that different evolutionary mechanisms may be involved in adaptation to As in admixed populations with Native-American ancestry historically exposed to high As levels.

## Materials and Methods

### Study Subjects

Subjects were invited to participate after signing an informed consent. Cases were recruited among patients treated for primary BC between 2013 and 2015 at the state-owned Hospital Regional de Antofagasta, which is the regional referral center for all major health problems occurring in northern Chile. The study was approved by the Ethics Committee of the Faculty of Medicine from Clínica Alemana Universidad del Desarrollo in Santiago. Based on this approval, it was subsequently authorized for execution by local authorities of the Hospital Regional de Antofagasta. Control subjects were individuals with no prior history of genitourinary malignancies. They were enrolled at the outpatient center of the same hospital and matched to the case patients by age (∼5 years) and sex. Epidemiologic and clinical data (demographics, smoking history, medical history, and occupational risk factors associated with exposure to aromatic amines [ORFs]) were collected during an in-person interview by trained nurses using a validated questionnaire. A blood sample was obtained at the same time. Smoking status was assigned according to current WHO-classification, with never smokers defined as individuals who had smoked none or <100 cigarettes during lifetime, and ever smokers as individuals who had smoked ≥100 cigarettes during lifetime.

Statistical significance between cases and controls of the following host characteristics was measured with the *χ*^2^ test of independence: sex, ORFs, mining workers, and smoking status. Differences in age between cases and controls were evaluated using a two-tailed *t*-test. *P *<* *0.05 was considered significant. Supplementary table 1, Supplementary Material online, shows statistical results for these comparisons. Mining workers and the remaining occupational risk factors considered together were considered as two separate categories.

### Sample Collection and Genotyping

Genomic DNA was purified from whole blood samples with the Blood Genomic DNA kit (Axygen, Corning, NY), and quantified using the Qubit dsDNA HS Assay (Life Technologies, Carlsbad, CA). All DNA samples analyzed passed the required quality controls for concentration, purity, and integrity. Genotyping was performed using the Affymetrix Human SNP 6.0 array (Affymetrix, Santa Clara, CA). Fluorescence was acquired on an Affymetrix Array Scanner 3000 7G, and quality control checks for each experiment were performed using the Affymetrix GeneChip Command Console 4.1.2 (AGCC) software. All identified variants were subjected to an additional gender-based control using the Genotyping ConsoleTM 4.0 (GTC) software, to verify the match between informed and genotyped gender.

APT version 1.15.1 and GCO2PLINK softwares were used to generate files with the entire genotyping and phenotype information and data were further analyzed with PLINK. SNPs that failed the filters for individual calling rate (mind <90%), marker calling rate (geno <90%), minor allele frequency (MAF ≤ 0.01), and/or Hardy–Weinberg equilibrium (>0.00001) were excluded from the analyses. All individuals were unrelated. After application of all filters, the initial number of 934,967 SNPs was reduced to 772,277.

### Local and Global Ancestry Inference

For the estimation of local and global ancestry in the Atacama cohort, we used LAMP-LD, which requires samples from the parental populations (Baran et al. 2012). We used reference panels for Native-American (*n* = 88), European (*n* = 911), and African (*n* = 229) populations (see supplementary table 3, Supplementary Material online, for details of the populations used in this study). Using an in-house Python script, the mean local ancestry was calculated for every SNP.

### Association between Genetic Variants and BC

Associations between genetic variants and the occurrence of BC were performed with Plink 1.9, using an additive genetic effects model. Data were adjusted for age, sex, global ancestry, ORFs, and tobacco smoking as described before. We did not find significant differences in the mean global Native-American ancestry between cases and controls (0.43 and 0.44, respectively; two sample *t*-test), suggesting that assortative mating on ancestry did not influence BC outcomes. Also, our association results are unlikely to be affected significantly by assortative mating on socioeconomic status, because volunteers were recruited at a public hospital were mostly people from low-middle socioeconomic levels (>90% of the total population) seek treatment. To correct for multiple comparisons across the 10 top hits detected by PBS_*n*__1_, we controlled the family wise error rate (FWER) at level *α* = 0.05 for a total of *n* = 10 ordered tests. The significance level for test *k* was set to *d*/*k*, where *d* is the solution to

(1) $$\prod_{k=1}^{n}\left(1-\frac{d}{k}\right)=1-\alpha .$$

### PBS*n*1 Test of Positive Selection

PBS*n*1 measures the amount of allele frequency change at each locus in a specific population since their divergence from two other populations. PBS*n*1 is a modified version of the PBS (Yi et al. 2010) that scales the statistic and avoids artificially high PBS values when differentiation is low or high between all populations (Crawford et al. 2017). PBS identifies local adaptation signatures in a particular population, by comparing the amount of allele frequency change at a specific locus in the history of that population since the divergence of two other populations. This test requires three populations as input. The subjects from Atacama (“ATA”) were regarded as the focal population. As secondary population, we chose a mix of native Maya, Nahua, Mixtec, and Tlapanec samples from Mesoamerica (MA) (Bigham et al. 2010). As third population, we chose a mix of Han Chinese and Japanese samples (EAS) from 1000 Genomes as a proxy population for the first humans who migrated into the American continent (see supplementary table 3, Supplementary Material online, for sample sizes). Nonpolymorphic sites in at least two populations were excluded (Amorim et al. 2017). The PBS value at each locus for ATA was calculated as follows:

(2) $${\text{PBS}}_{\text{ATA}}=T_{\text{ATA};\text{MA}}+T_{\text{ATA};\text{EAS}}–T_{\text{MA};\text{EAS}}/2,$$

where *T_X_*_;__*Y*_ is an *F*_ST_—derived metrics obtained by comparing populations *X* and *Y* defined as

(3) $$T_{X;Y}=–\;\text{log}(1–{\text{FST}}_{X,Y}).$$

We used Hudson’s *F*_ST_ estimator to account for differences in population sizes (Hudson et al. 1992). SNP-wise *F*_ST_ as well as global *F*_ST_ were computed using a published R script (Di Gaetano et al. 2014). PBS_*n*__1_ was calculated as follows:

(4) $${\text{PBS}}_{n1}={\text{PBS}}_{1}/1+{\text{PBS}}_{1}+{\text{PBS}}_{2}+{\text{PBS}}_{3},$$

where PBS_1_ is the PBS calculated with ATA as the focal group, PBS_2_ is the PBS calculated with MA as the focal group, and PBS_3_ is the PBS calculated with EAS as the focal group (Crawford et al. 2017). Because we aimed at detecting Native-Americans variants underlying local adaptation, we corrected the allele frequencies in ATA for admixture with Spaniards similarly as described previously (Huerta-Sánchez et al. 2013), using

(5) $$f_{\text{ATA}}^{*}=f_{\text{ATA}}–\alpha f_{\text{SPN}}/1–\alpha .$$

Here, $f_{\text{ATA}}^{*}$ and *f*_ATA_ represent the allele frequency at a locus in ATA before and after admixture, respectively. *f*_SPN_ represent the allele frequency at the same locus in Spaniards from POPRES (supplementary table 3, Supplementary Material online), a proxy population for the 16th century Spanish population that admixed with Native Americans. *α* represents the proportion of ancestry derived from Spaniards at that locus and was inferred with LAMP-LD as explained before.

### Estimation of the Distribution of Maximum PBS_*n*__1_ Values

To estimate the distribution of the maximum value of a sample of PBS_*n*__1_, we generated a random sample of 1,000 values from the full set of 402,287 valid PBS_*n*__1_ values. From this sample, we estimated the maximum value and the mean value. We repeated this 1,000 times to obtain 1,000 mean values and 1,000 maximum values. The estimates of the distribution of each of the 1,000 samples are shown in supplementary figure 2, Supplementary Material online. We fitted a generalized extreme value distribution to the maximums using the R package extRemes. With this cumulative distribution, we obtained that the probability of observing a value ≥0.34 (the value of the tenth highest score on PBS_*n*__1_) is 0.014. The probability of observing a value of PBS_*n*__1_ ≥ 0.45 (the highest PBS_*n*__1_ value) is 0.0034. Supplementary figure 3, Supplementary Material online, shows the estimate of the distribution.

### Estimation of Background Selection

We obtained background selection (BGS) coefficients (*B*) for each variant rate (McVicker et al. 2009). *B* indicates a reduction of the effective population size (*N*_e_) at neutral sites as a function of their recombination distance from conserved and exomic loci, the strength of purifying selection at these loci, and the deleterious mutation rate (McVicker et al. 2009; Torres et al. 2018). Positions for *B* were lifted over from hg18 to hg19 using the UCSC liftOver tool.

### Overlap between Loci Selected by PBS_*n*__1_ and Low BC Association *P* Values

One thousand random samples of *P* values of size *n* = 100 were generated. *P* values from the samples of 100 were matched to have “similar” characteristics in recombination rate and derive allele frequency from the SNPs with the highest 100th scores on PBS*n*1. We defined “similar” as the intervals defined by 5% of values above and below the observed ones in recombination rate and derived allele frequency. The randomization test described in the Results section was performed using R scripts. The means of BC association *P* values from the random and PBS_*n*__1_ selected loci were compared using a Welch two sample one-tailed *t*-test. A *P* value <0.05 was considered significant.

### Deviations from Mean Genome-Wide Local Ancestry

We used *t*-tests to identify SNPs with an excess of Native-American ancestry. At each SNP, we compared its Native-American ancestry proportion to the genome-wide ancestry proportion, denoted by *p*_0_ = 0.44, by performing a statistical hypothesis test:

(6) $$H_{0}:p_{i}=p_{0}\;\text{vs}\;H_{1}:p_{i}\neq p_{0}$$

at each SNP, where *p_i_* represents the Native-American ancestry at the *i*th SNP. To do that, we assumed that *X_ij_* is a random variable distributed Binomial with parameters *n *=* *2 and *p_j_*, where *X_ij_* takes values in {0, 1, 2} and represent the number of Native-American ancestry in SNP *j* and individual *i*. *p_j_* is the proportion of Native-American ancestry at SNP *j*. We used the asymptotic results of the maximum likelihood estimator for *p_j_*, to assume a normal distribution on this estimator and to design our hypothesis test. Variants achieving a significance threshold of *P *<* *5 × 10^−5^ were regarded as selected (Bhatia et al. 2014).

### Variant Annotations

All variant annotations used in this study correspond to the GRCh37 (hg19) assembly. They include the Sequence Ontology (SO) consequence type, Gencode biotypes and Ensembl feature types and were retrieved using the web tool VEP from Ensembl (McLaren et al. 2016). Each variant was classified into one of the following SO consequence types: *intron, intron/noncoding transcript*, *noncoding transcript exon/noncoding transcript*, *missense*, *synonymous*, *3 prime UTR*, *5 prime UTR and intron/NMD transcript*, *intergenic*, *regulatory region*, and *downstream-gene and upstream-gene variants*. Upstream and downstream variants were defined as those variants located 10-kb upstream or downstream of the gene, respectively. Intergenic variants were defined as those located >10-kb upstream or downstream of the closest gene.

## Results

### Detection of Preadmixture Native-American Adaptation Signals to As Exposure

**Fig. 2.**
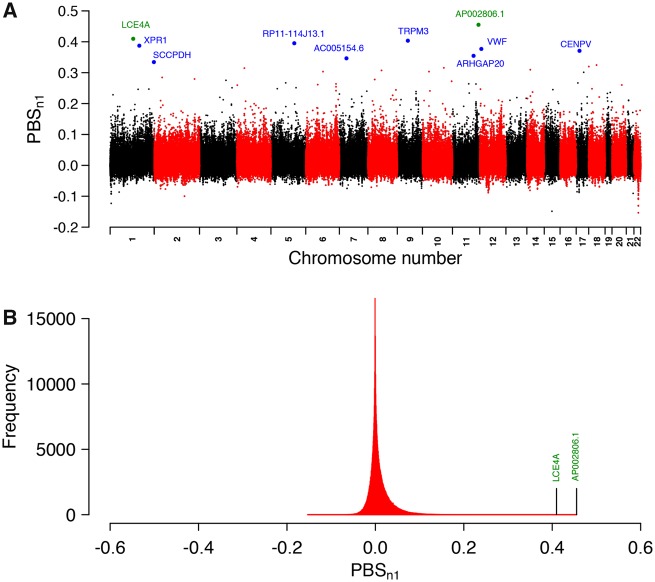
—PBS_*n*__1_ scores across the genome. (*A*) Manhattan plot showing the PBS_*n*__1_ scores along autosomic chromosomes. Blue and green dots represent selected variants with their associated genes. Green dots represent the two strongest hits. (*B*) Distribution of PBS_*n*__1_ scores. Marked positions correspond to genes associated with the two strongest hits.

PBS*n*1 captures SNPs where allele frequencies are especially differentiated in a focal population when compared with two other populations (Yi et al. 2010; Crawford et al. 2017). We used this test to detect highly differentiated loci in the Andean ancestors of the admixed Atacama people (the focal population). We chose a mix of native individuals from Mesoamerican populations (Bigham et al. 2010) as a second related population in order to capture differentiated adaptation signatures in the Atacama people that occurred after their divergence from Mesoamericans ∼12,000 ya (Harris et al. 2018). We chose a mix of native individuals from East Asia as the third population, which represents a proxy of the first humans who migrated from northeast Siberia to the American continent through Beringia at least ∼23,000 ya (Nielsen et al. 2007). The allele frequencies in the admixed Atacama people were corrected for admixture with Europeans similarly as described previously (Huerta-Sánchez et al. 2013), using modern Spaniards as a proxy for the European ancestral population of the Atacama people. This pseudounadmixed population is called “Atacamas” from now on. Genome-wide genetic differentiation was very low between Atacamas and Mesoamericans (*F*ST = 0.026), higher between Mesoamericans and East Asians (*F*ST = 0.094) and the highest between Atacamas and East Asians (*F*ST = 0.111), indicating that *F*ST-based tests like PBS*n*1 have good power to capture positive selection signals (Crawford et al. 2017). The top ten SNPs with the highest PBS*n*1 scores are shown in table 1. We refer hereafter to these variants as “selected.” Figure 2*A* shows the PBS*n*1 scores across the genome, highlighting genes associated with selected variants. The strongest signals are mentioned in the Discussion section. Figure 2*B* shows the PBS*n*1 scores distribution, which exhibits a positive long tail, highlighting genes associated with the two strongest hits.

**Table 1:** Top Variants Selected by PBS_*n*__1_

Chr	SNP ID	*f*_ATA_	$f_{\text{ATA}}^{*}$	*f*_MA_	*f*_EAS_	PBS_*n*__1_	*B*	Gene	Conseq.	(KB)
3	rs2276440	0.454	0.910	0.012	0.076	0.4551	0.949	AP002806.1	DS	9.8
1	rs11205084	0.413	0.903	0.051	0.123	0.4096	0.778	LCE4A	US	7.6
9	rs1329776	0.5	0.896	0.051	0.123	0.4035	0.891	TRPM3	IN	0
5	rs17586072	0.429	0.958	0.205	0.128	0.3952	0.888	RP11-114J13.1	IN-NC	0
1	rs3002116	0.527	0.819	0.013	0.057	0.3873	0.594	XPR1	IN	0
12	rs17419697	0.5	0.238	0.988	0.998	0.3768	0.797	VWF	IN	0
17	rs3095168	0.546	0.087	0.795	0.921	0.3707	0.666	CENPV	US	3.3
11	rs17112293	0.391	0.857	0.205	0.117	0.3545	0.850	ARHGAP20	IG	414
7	rs10234832	0.470	0.905	0.294	0.005	0.3464	0.733	AC005154.6	NC	0
1	rs10924824	0.664	0.978	0.308	0.252	0.3342	0.858	SCCPDH	DS	0.3

Note.—Chr, chromosome; *f*_ATA_ and $f_{\text{ATA}}^{*}$, derived allele frequency in the admixed and pseudounadmixed populations from Atacama, respectively. *f*_MA_ and *f*_EAS_, derived allele frequency in Mesoamericans and East Asians, respectively; PBS_*n*__1_, score; *B*, background selection coefficient; Conseq., SO consequence type. Abbreviations: DS, downstream; US, upstream; IN, intron; IN-NC, intron/noncoding transcript; IG, intergenic; NC, noncoding transcript. KB: kb from transcript.

To assess how unlikely is to obtain the top PBS_*n*__1_ scores, we estimated the distribution of the maximum of a sample based on the full set of PBS_*n*__1_ measurements and extreme value theory (see a detailed description of the test in the Materials and Methods section). We obtained that the probability of observing a value ≥0.34 (the value of the tenth highest PBS_*n*__1_ score) is 0.014. The probability of observing a value of PBS_*n*__1_ ≥ 0.45 (the highest PBS_*n*__1_ value) is 0.0034. Supplementary figures 2 and 3, Supplementary Material online, show the distribution plots.

In order to evaluate whether accelerated allele frequency differentiation of selected variants is due to BGS (Cruickshank and Hahn 2014), we obtained BGS coefficients (*B*) for selected variants. *B* values represent the reduction of neutral genetic diversity at a particular locus along the genome that is caused by BGS (McVicker et al. 2009). *B* ranges from 0 to 1, with *B *=* *0 indicating an almost complete removal of genetic diversity due to BGS, and *B *=* *1 indicates no effect of BGS on neutral genetic diversity. The mean *B* score of selected variants was 0.78, and the selected variant with the lowest *B* score had *B *=* *0.594 (table 1 and supplementary fig. 4, Supplementary Material online). This suggests that high allele frequency differentiation of the selected SNPs is not due to BGS.

### Associations between Selected Variants and BC in Subjects Exposed to As

Several organs and biological pathways have been hypothesized to be affected by adaptation to high As levels in humans, including the liver, the cardiovascular system and the respiratory system (Schlebusch et al. 2015). However, hitherto no study has evaluated such hypotheses. We evaluated whether adaptation to As may have affected the urinary bladder. We performed association tests between the ten variants selected by PBS*n*1 and BC occurrence in this cohort. This approach has proven successful in identifying variants and phenotypes underlying adaptation to extreme environments when sample sizes are small (Yi et al. 2010; Fumagalli et al. 2015; Ilardo et al. 2018), by alleviating the burden of multiple testing used in genome-wide association studies (GWAS) (Ilardo et al. 2018). We compared 92 cases who were treated for primary BC between 2013 and 2015 versus 93 controls with no BC diagnosis in these years. We adjusted for global ancestry, age, sex, and tobacco smoking. Tobacco is the most important risk factor for BC, being responsible for at least 50% of all cases (Freedman et al. 2011). Because ORFs are estimated to account for up to 10% of BC cases (Cumberbatch et al. 2015), we also adjusted the results for subjects who worked in at least one of the following activities: printing, hairdressing, mining, chemical industry, or rubber industry. No significant difference was found for age, sex, smoking status, or ORFs between cases and controls (supplementary table 1, Supplementary Material online). We accounted for multiple testing across selected SNPs by controlling the FWER at level *α* = 0.05 for a total of ten ordered tests. Here, a decreasing significance threshold was assigned to each selected locus according to their order in the ranking of PBS*n*1 scores (see Materials and Methods section for a description of the test). Table 2 shows the association *P* values and corresponding significance thresholds. The strongest association was achieved by the *rs11205084* variant of *LCE4A-C1orf68* (*P *=* *0.012; significance threshold = 0.0087; table 2).

**Table 2:** Association between Loci Selected by PBS_*n*__1_ and BC Occurrence

SNP ID	*P* Value	Significance Threshold
rs2276440	0.060	0.01743
rs11205084	**0.012**	0.0087
rs1329776	0.040	0.0058
rs17586072	0.901	0.0043
rs3002116	0.087	0.0034
rs17419697	0.046	0.0029
rs3095168	0.313	0.0024
rs17112293	0.640	0.0021
rs10234832	0.446	0.0019
rs10924824	0.140	0.0017

Note.—Shown are SNP ID, the association *P* value, and the significant threshold after controlling the FWER. The strongest association is indicated in bold.

**Fig. 3.**
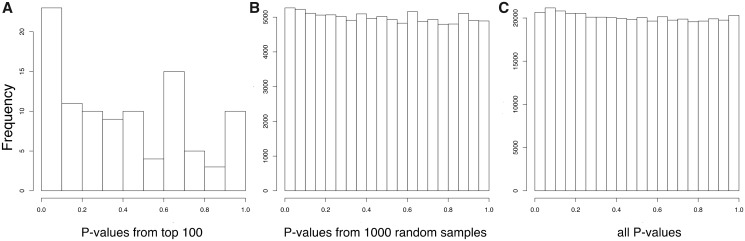
—Distribution of BC association *P* values among SNPs with highest PBS*n*1 scores. (*A*) Histogram showing observed *P* values for the top 100 PBS*n*1 SNPs. (*B*) Histogram of 1,000 random samples of *P* values of size *n* = 100. (*C*) Histogram of all BC association *P* values from the data set.

In order to assess whether there is enrichment in low association *P* values for BC among the top loci selected by PBS*n*1, we compared the distribution of all *P* values with the distribution of *P* values obtained from 1) the top 100 SNPs with highest PBS*n*1 scores and 2) 1,000 random samples of size n = 100. Each of the 1,000 random samples of size *n* = 100 were obtained by randomly picking *P* values from SNPs with similar characteristics in recombination rate and derive allele frequency from the top SNPs with highest PBS*n*1 scores. We explain in detail the sampling procedure in the Materials and Methods section. Figure 3*A* shows the distribution of *P* values from the 100 SNPs with highest PBS*n*1 scores, figure 3*B* shows the distribution of all *P* values and figure 3*A* shows the distribution of *P* values obtained from the random samples. These figures show an enrichment of small *P* values among the 100 SNPs with highest PBS*n*1 scores. We also calculated the mean BC association *P* value among loci with the top 5, top 10, top 25, top 50, and top 100 PBS*n*1 scores between the corresponding observed and random sets of *P* values. We found significant differences between the means of selected and random sets of BC association *P* values among the top 10, top 50, and top 100 sets of values, but not among the top 5 or top 25 sets of values. Supplementary table 2, Supplementary Material online, shows the summary statistics of the corresponding *t*-tests.

### Postadmixture Adaptation Facilitated by Native-American Variants

**Fig. 4.**
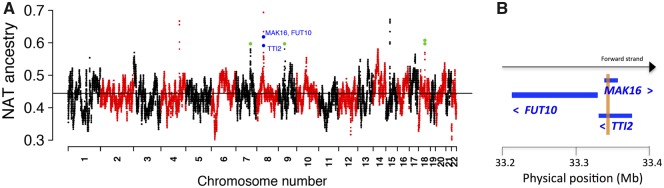
—Mean local Native-American ancestry along the genome. (*A*) Green dots represent SNPs with significant deviations in the mean local Native-American ancestry over the genome-wide mean in the whole cohort and in controls considered separately, but not in cases considered separately. Blue dots pinpoint the strongest hits (*P *=* *1.3 × 10^−9^), which are associated with the *MAK16/FUT10/TTI2* gene cluster of chromosome 8. The black horizontal line represents the genome-wide mean of the local Native-American ancestry of the complete cohort. (*B*) Simple representation of the genetic map of the *MAK16/FUT10/TTI2* gene cluster (for a detailed genetic map see supplementary fig. 6, Supplementary Material online). The light brown vertical frame shows the overlapping of regions between MAK16 and TTI2. Blue arrowheads indicate the transcriptional orientation of the genes.

Adaptive selection can act through gene flow between populations after an admixture event has occurred (Jeong et al. 2014). One way to detect it is to identify variants with significant deviations of the mean local ancestry from the genome-wide mean (Bryc et al. 2010; Bhatia et al. 2014; Jeong et al. 2014). In order to address whether the Atacama population inherited Native-American adaptation signals after the admixture between Native Americans and Europeans, we looked for deviations in the Native-American ancestry in the whole cohort. Local ancestry inference was performed with LAMP-LD (Baran et al. 2012), which uses samples from Native Americans, Europeans and Africans as proxies for ancestral populations (Eyheramendy et al. 2015). LAMP-LD identified at each individual in the study population, chromosomic regions with Native-American, European, or African ancestry. In our cohort, LAMP-LD estimated genome-wide local ancestry means of 0.441 (Native American), 0.521 (European), and 0.037 (African). At each SNP, the mean Native-American ancestry was compared with the genome-wide Native-American ancestry mean. Using *t*-tests, we evaluated the hypotheses *H*0: μ_NAT;__*i*_ = 0:441 versus *H*1: *μ*_NAT;__*i*_ > 0:441 and *H*0: *μ*_EUR;__*i*_ = 0:521 versus *H*1: *μ*_EUR;__*i*_ > 0:521, at each variant *i*. We detected 1,687 SNPs achieving the significance threshold of 5 × 10^−5^ recommended for recently admixed populations (Bhatia et al. 2014). Next, we excluded variants enriched in Native-American ancestry unrelated to the toxic effects As induces in the bladder. Thus, we looked for SNPs with significant Native-American ancestry deviations in the whole cohort as well as in BC controls considered separately, but which did not show significant deviations in BC cases considered separately. In this way, we obtained 188 SNPs. Figure 4*A* shows the mean local Native-American ancestry across the genome and highlights these 188 SNPs. Supplementary file 1, Supplementary Material online, shows the corresponding *P* values as well as annotations for these SNPs. Among these 188 variants, the strongest deviation in the Native-American ancestry was equally achieved by 31 SNPs in a region from chromosome 8 encompassing *MAK16* and *FUT10* genes (*P *=* *1.3 × 10^−9^), including a missense variant in *MAK16* (supplementary file 1, Supplementary Material online). Supplementary figure 5, Supplementary Material online, shows global ancestry proportions for each individual, estimated using LAMP-LD.

Further, we found that the MAK16 variants were in high LD with multiple variants in *FUT10* (*r*2 = 0.90–0.95) as well as in the *TTI2* gene (*r*2 = 0.9–1) (data queried from Ensembl), indicating that these three genes share a common haplotype. Indeed, exon 10 of *MAK16* overlaps exons 6 and 7 of *TTI2* (fig. 4*B*). Supplementary figure 6, Supplementary Material online, shows the detailed genetic map of these genes. We also found that the proteins encoded by *MAK16*, *FUT10*, and *TTI2* are expressed in several cancer types, including urothelial cancer. Indeed, these proteins showed high/medium expression in 25%, 80%, and 100% of urothelial cancer patients tested, respectively (supplementary fig. 7, Supplementary Material online); data obtained from the Human Protein Atlas (Uhlén et al. 2015).

## Discussion

Populations with Andean ancestry have been exposed for millennia to high As levels in water and present genetic signatures of adaptation to As in their genomes. For instance, studies in populations from northern Chile and Argentina found adaptations likely acting by improving the As-detoxifying activity of the *AS3MT* enzyme (Eichstaedt et al. 2015; Schlebusch et al. 2015; Apata et al. 2017). Some of these studies identified additional candidate genes targeted by As-driven adaptation, but their potential roles in this adaptation is unknown. It is also not known which organs are mostly affected by As toxicity, whose dysfunction arguably decreases fitness in these populations. For instance, it was suggested that adaptation to As could act by targeting the toxic effects it produces in the lung, immune cells, liver, and/or cardiovascular system (Schlebusch et al. 2015). In the present study, we addressed whether adaptation to As exposure could have affected the urinary bladder. To our knowledge, this is the first study that experimentally evaluates the downstream effects of As-driven positive selection on a specific organ or tissue system.

The population from the Atacama Desert in northern Chile is a unique model to study how adaptation to As exposure could have an effect on the urinary bladder. First, their local Native-American ancestors were of Andean ancestry and were exposed to high As levels (∼120 μg/l) for at least 5,000 years (Byrne et al. 2010; Latorre et al. 2013; Yáñez et al. 2015), enabling positive selective sweeps to act over hundreds of generations. Second, this selective pressure substantially increased in magnitude (up to ∼600 μg/l) during 1958–1971 in this population, producing significantly higher BC incidence and BC-specific mortality rates at present when compared with other Chilean regions (Fernandez et al. 2012). Third, we were able to recruit a case–control cohort with As-induced BC that was exposed to As ∼5 decades ago, enabling us to study the effects of adaptation to As over BC, a relatively rare phenotype.

We used the PBS*n*1 test to identify adaptation signatures underwent by the Andean ancestors of the Atacama people after their split from Mesoamericans ∼12,000 ya (Harris et al. 2018). The top selected locus was *rs2276440* and is located 9.8-kb downstream of the *AP002806.1* miRNA, whose biological functions have not been described. The second top selected locus was *rs11205084*, which showed the strongest association with BC occurrence (*P *=* *0.012) among loci selected by PBS*n*1. This variant is located 7.7-kb upstream of the *LCE4A-C1orf68* gene, which is involved in the barrier function of the epithelium (Kumar et al. 2014). Interestingly, urothelial BC originates exclusively in the urothelial epithelium (urothelium) (Chung et al. 2013). *LCE4A-C1orf68* is a member of the Late Cornified Envelope (LCE) gene cluster. Of notice, the transcription of some LCE genes is activated by direct binding of p53 protein to LCE enhancers. p53 is one of the most important tumor suppressor genes in human carcinogenesis (Deng et al. 2014). Besides, *LCE4A-C1orf68* variants are associated with susceptibility to infectious diseases, such as tuberculosis (Tian et al. 2017) and candidaemia (Kumar et al. 2014), as well as with autoimmune diseases, such as rheumatoid arthritis (Uddin et al. 2011) and psoriasis (Zhang et al. 2009). Moreover, the risk of BC increases significantly after autoimmune diseases, including rheumatoid arthritis and psoriasis (Liu et al. 2013). These observations suggest that *LCE4A-C1orf68* might be involved in the immune barrier exerted by the urothelium (Lazzeri 2006) or other relevant epithelial tissues against dysregulated immune factors during carcinogenesis. Among loci selected by PBS*n*1, we also found a variant mapping the *VWF* gene (table 1), which is associated with traits of possible importance for As resistance. *VWF* encodes a key protein involved in coagulation, by facilitating adhesion of platelets to the endothelium of blood vessels. Also, high *VWF* levels are a well-known risk factor for arterial thrombosis (heart attack) (Sanders et al. 2013). Interestingly, a close relation between As concentration in the heart and coronary thrombosis has been reported in the Antofagasta region of northern Chile (Pizarro et al. 2012). Thus, loci selected by PBS*n*1 could also underlie adaptation to the detrimental effects As produces in the heart.

Our randomization results on BC association *P* values among loci detected by PBS*n*1 (fig. 3) show a clear enrichment in low *P* values among top PBS*n*1 loci, suggesting that polygenic adaptation to As affected the function of the urinary bladder in this population. Some of these loci might reflect a pleiotropic effect that resulted from adaptation to other cancers induced by high As exposure, such as lung and kidney cancers (Marshall et al. 2007; Ferreccio et al. 2013; Chen et al. 2018). Besides, we expect that some loci selected by PBS*n*1 are related to other environmental factors (e.g., local infectious pathogens) that were unique to the ancestral population of Atacamas which split from Mesoamericans ∼12,000 years ago (Ilardo et al. 2018).

The strongest deviations in the mean local Native-American ancestry captured by the ancestry-enrichment test were equally achieved by 31 SNPs in a region from chromosome 8 mapping *MAK16* and *FUT10* genes (*P *=* *1.3 × 10^−9^). These genes are in high LD with *TTI2*, which shares common exons with *MAK16* and was also among the genes selected by the ancestry-enrichment test (supplementary file 1, Supplementary Material online). *MAK16* encodes a ribosomal protein with an important function in the biogenesis of eukaryotic ribosomes (Kater et al. 2017). Mutations affecting ribosomal structure and function have a causal relation with the development of many cancers (Pelletier et al. 2018). In particular, mutations in *MAK16* induce the arrest of the cell cycle at G1 phase, during which the cell synthesizes mRNA and proteins in preparation for cell division. Remarkably, As exposure also induces the arrest of the cell cycle at G1 and G2M phases (Flora 2011). Several observations also support a likely involvement of *FUT10* and *TTI2* in cell cycle as well as cancer. *FUT10* encodes a fucosyltransferase whose overexpression increases self-renewal of stem cells, whereas its suppression induces the differentiation of these cells (Kumar et al. 2013). Also, fucosylation is suggested to mediate tumor cell adhesion and proliferation in certain cancers (Carrascal et al. 2018). Thus, *FUT10* may alter the cell cycle during tumorigenesis. *TTI2* encodes a subunit of a master regulator complex of phosphoinositide-3-kinase-related protein kinase abundance and DNA damage response (Langouët et al. 2013). In turn, phosphoinositide-3-kinase-related protein kinases are involved in many signaling pathways, including those controlling cell-cycle progression (Abraham 2001). Taken together, these observations suggest that one or more Native-American variants in this gene cluster may be related with adaptation against As exposure following admixture, possibly by targeting cell-cycle proteins whose dysregulation produces cancer. However, due to the high LD and the fact that our data were from a SNP chip, it is difficult to pinpoint causative SNP(s) driving selection (Fumagalli et al. 2015). Interestingly, the ancestry-enrichment test identified significant variants that have been associated with physiological responses to cigarette smoke, which contains considerable levels of this xenobiotic and is a well-known risk factor for BC (Chung et al. 2013). One of them, *rs848353* (*P *=* *8.7 × 10^−8^) maps the *AC004014.3* gene (supplementary file 1, Supplementary Material online) and has been associated by GWAS with nicotine dependence, number of cigarettes smoked per day and heaviness of smoking in East Asians (Yoon et al. 2012). The ancestry-enrichment test identified another variant, *rs984655* (*P *=* *2.8 × 10^−8^), located in the *TPMTP1* gene (supplementary file 1, Supplementary Material online). Loci in this gene have been GWAS-associated with impaired pulmonary function in smokers (Lutz et al. 2015). The observation that variants detected by the ancestry-enrichment test map genes previously associated with physiological responses to cigarette smoke suggests postadmixture adaptation to As exposure.

We did not find an overlap between the hits detected by PBS*n*1 and the ancestry-enrichment test. This is not surprising, because these tests detect different kinds of adaptation signatures. PBS*n*1 is more suitable for detecting (preadmixture) adaptation driven by selective sweeps when selection is acting on standing variation (Yi et al. 2010). On the other hand, the ancestry-enrichment test detects postadmixture adaptation facilitated by gene flow, which is an evolutionary force different than selective sweeps acting on standing variation, and which closely resembles adaptive introgression (Bhatia et al. 2014; Jeong et al. 2014). Further, admixture with Europeans strongly modified—mostly decreased—the frequency of the Native-American adaptive alleles, as shown in table 1 (columns *f*_ATA_ and $f_{\text{ATA}}^{*}$). Arguably, if the same levels of As exposure are maintained over the admixed population, it would take several generations for that allele to arise into high frequency again. Among Chileans, admixture took place ∼10 generations ago (Eyheramendy et al. 2015), a relatively short time for adaptive alleles to recover the frequencies of the pseudounadmixed population. Future demographic inference analyses would be needed to test this hypothesis. These observations suggest that different sets of genes may play a role in adaptation to As depending on the genetic mechanisms behind the adaptation forces detected by the two tests.

The main native population from northern Chile is Aymara. However, until the annexation of a big part of the Atacama Desert by Chile from Bolivia in 1884 following the Pacific War, the major native populations in the Antofagasta region were Atacameño. Thereafter, continuous admixture events resulted in a present-day population in this region having ∼46% Native-American, ∼50% European, and ∼4% African ancestry (Eyheramendy et al. 2015). The lack of published genomes from extinct native populations from Atacama makes it difficult to identify the source population of the adaptation signatures selected in our cohort. A further constraint is that genetic adaptation to As in a population can exhibit significant subpopulation differences. For instance, an Aymara population from northern Chile historically exposed to high As levels in water has adaptive genetic variants in the As-related *AS3MT* gene. In contrast, a closely located and related population unexposed to As lacked these adaptations (Apata et al. 2017). It is likely that the most recent native ancestors of the modern Atacama population ultimately inherited As-related adaptation variants from an ancient Andean population ancestral to As-resistant populations from Chile and Argentina (Apata et al. 2017). In addition, the admixed Atacama population might have undergone further As-related adaptations due to the high As levels present in water (117–600 μg/l, in contrast to the 10 μg/l currently recommended by WHO for drinking water (Fernandez et al. 2012) before the incorporation of cleaner water sources in 1971 (fig. 1).

In summary, the results of this study contribute to a better understanding of the genetic factors underlying physiological adaptation in populations with Native-American ancestry historically exposed to As in water.

## Data Access

The data generated in this study can be found in the following link: http://200.54.225.60/∼boris/mfernandez.

## Supplementary Material

Supplementary data are available at *Genome Biology and Evolution* online.

## Supplementary Material

evz172_Supplementary_Data
