# Supplementary material for: Adaptation to Extreme Environments in an Admixed Human Population from the Atacama Desert
Source: Genome Biol Evol. 2019 Aug 6;11(9):2468–79. doi: 10.1093/gbe/evz172 (PMC6733355; doi:10.1093/gbe/evz172)
Supplement: evz172_Supplementary_Data [file evz172_supplementary_data.zip › Supplemental_Material.pdf]

SUPPLEMENTARY FILES

Supplementary Figures

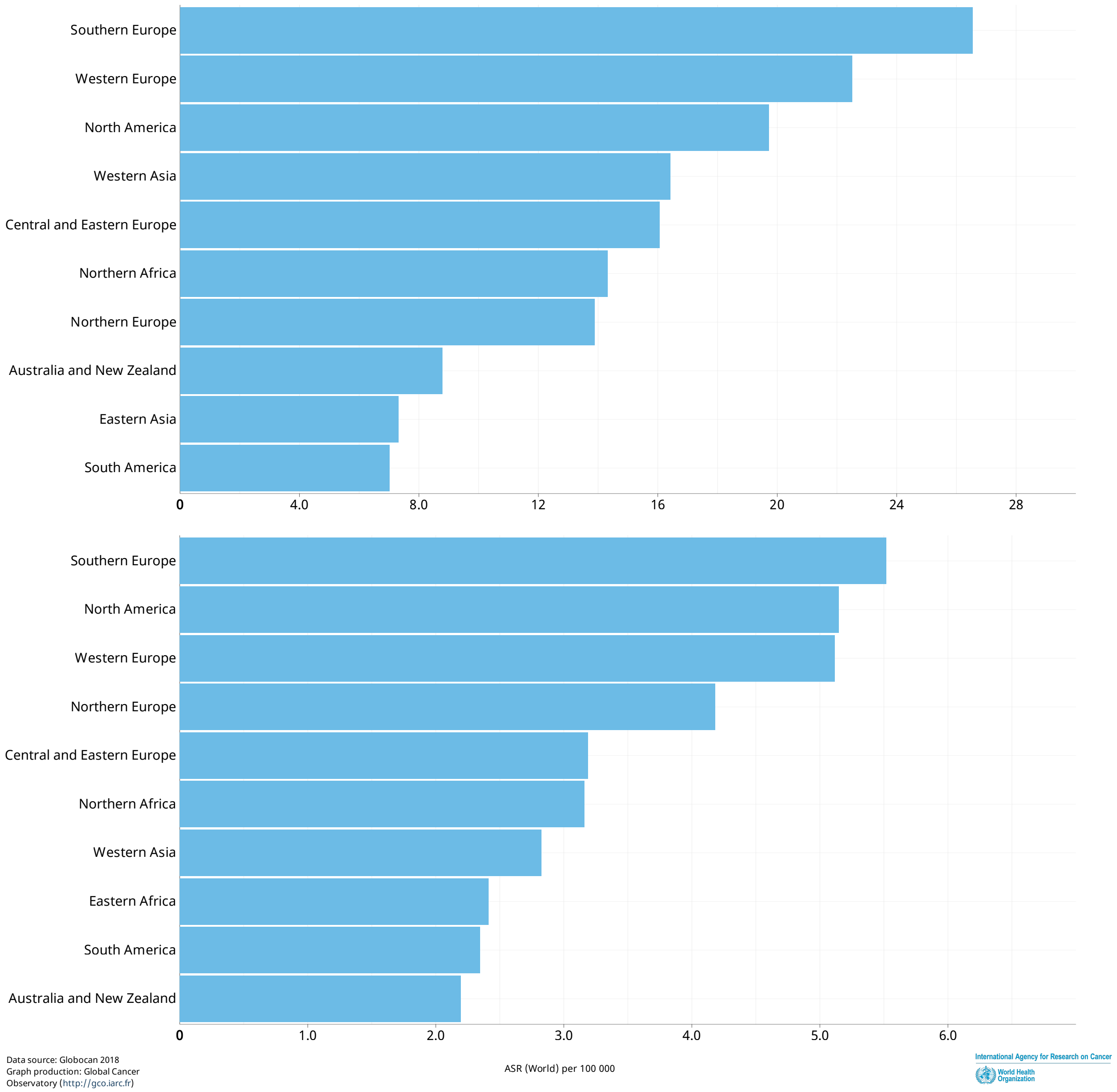

Supplementary Figure 1. Estimated incidence rates of BC across the world. Age-standardized BC incidence rates (ASR) across 10 world regions among males (upper panel) and females (lower panel). ASR is shown in units of number of affected subjects per 100,000 individuals. This image was retrieved from the International Agency for Research on Cancer webpage (`https://www.iarc.fr/`)

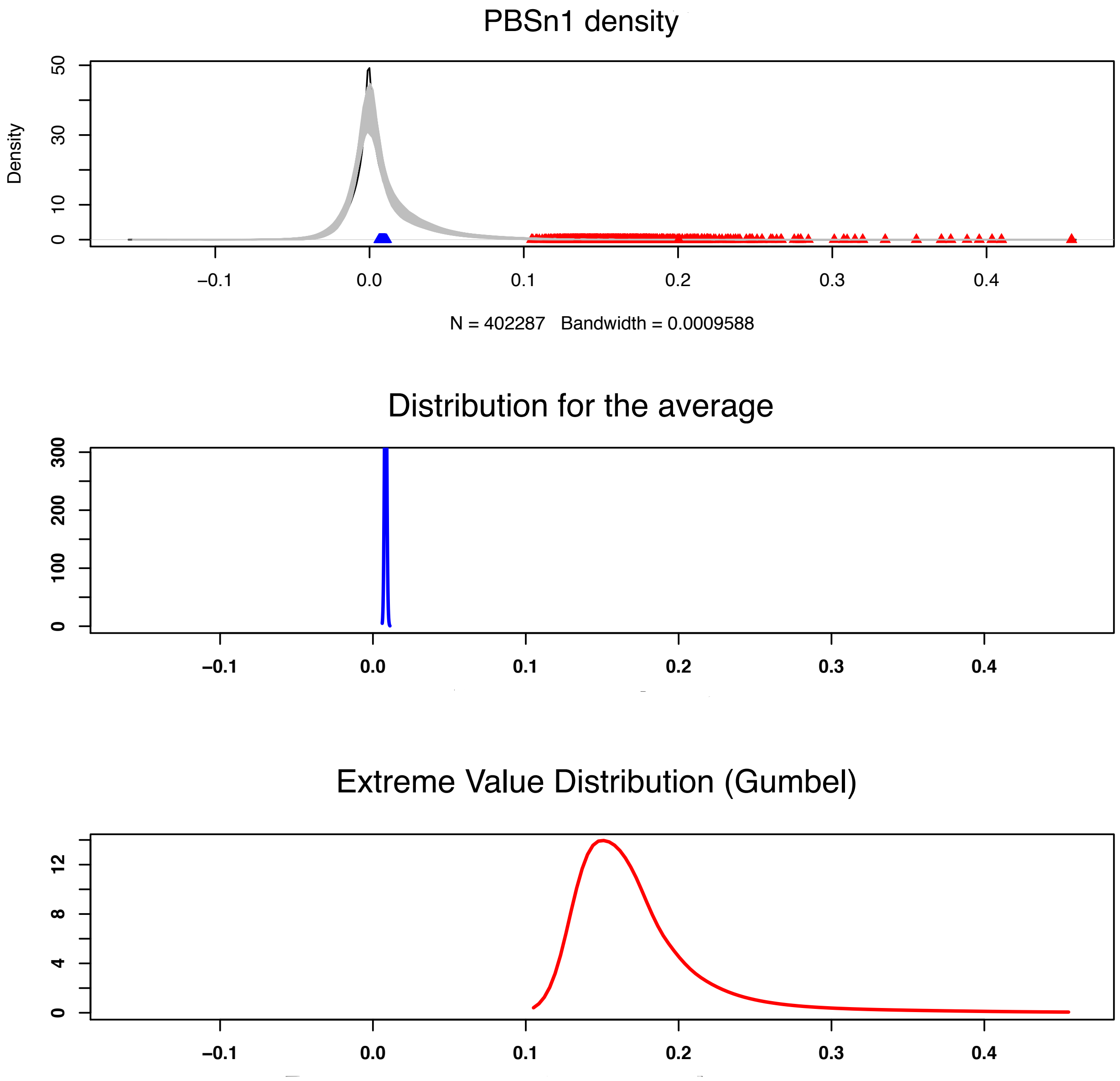

Supplementary Figure 2. Distribution of maximum and mean PBS_{n1} scores from 1000 random samples. Upper panel. Grey lines represent the estimate of the distribution of each of the 1000 samples; red triangles and blue dots represent the maximum and mean values obtained in each sample, respectively. Middle panel. Distribution of the means. Lower panel. Distribution of the maximums.

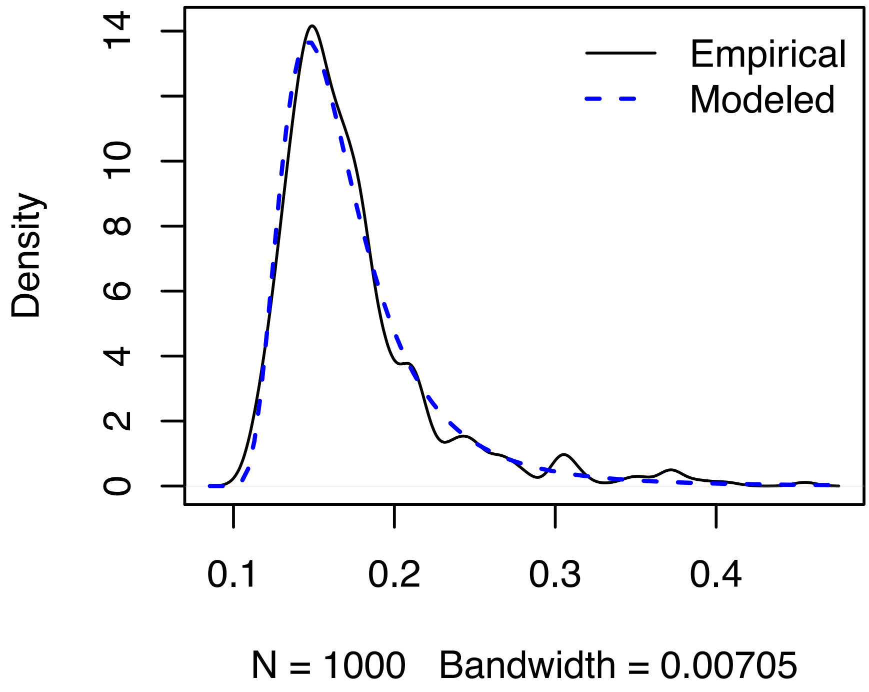

Supplementary Figure 3. Density plot showing the distribution of empirical and modeled PBS$_{n1}$ scores.

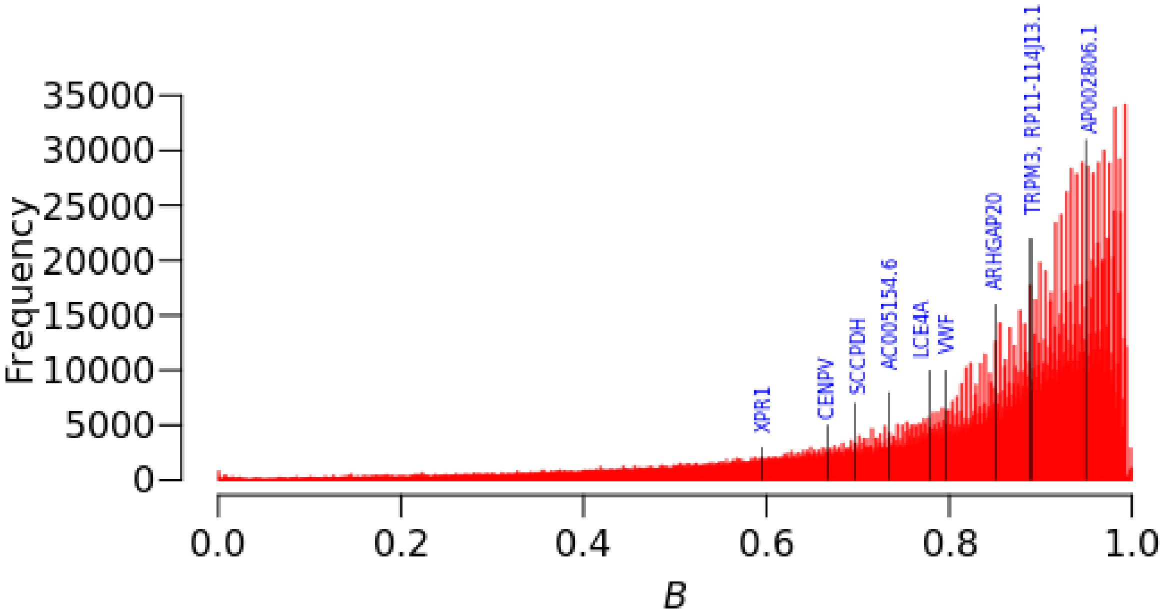

Supplementary Figure 4. Distribution of *B* scores across the genome. Genes associated with variants selected by PBS_{n1} are highlighted in blue.

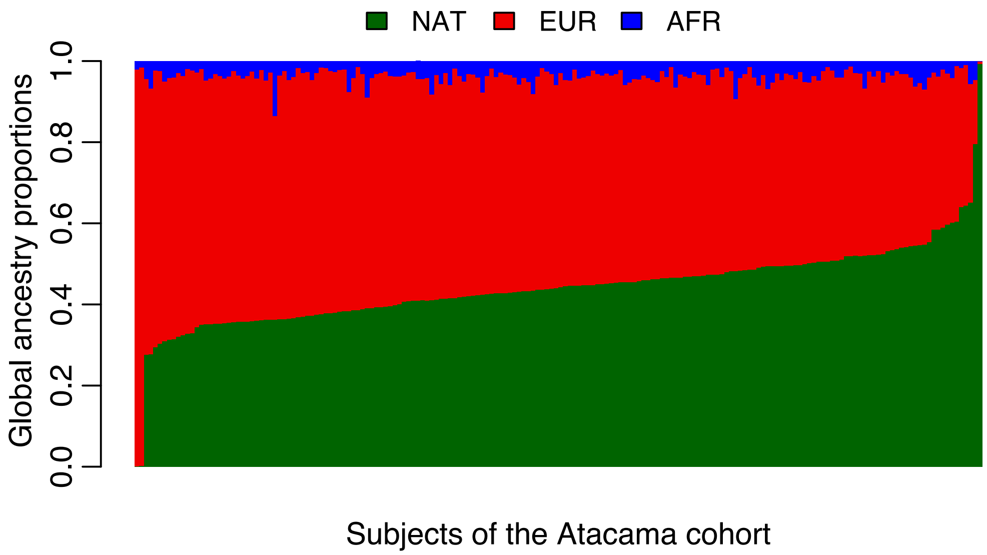

Supplementary Figure 5. Individual global ancestry estimates. Samples are ordered from left to right in ascending order of Native-American ancestry proportion. NAT: Native-American, EUR: European, AFR: African.

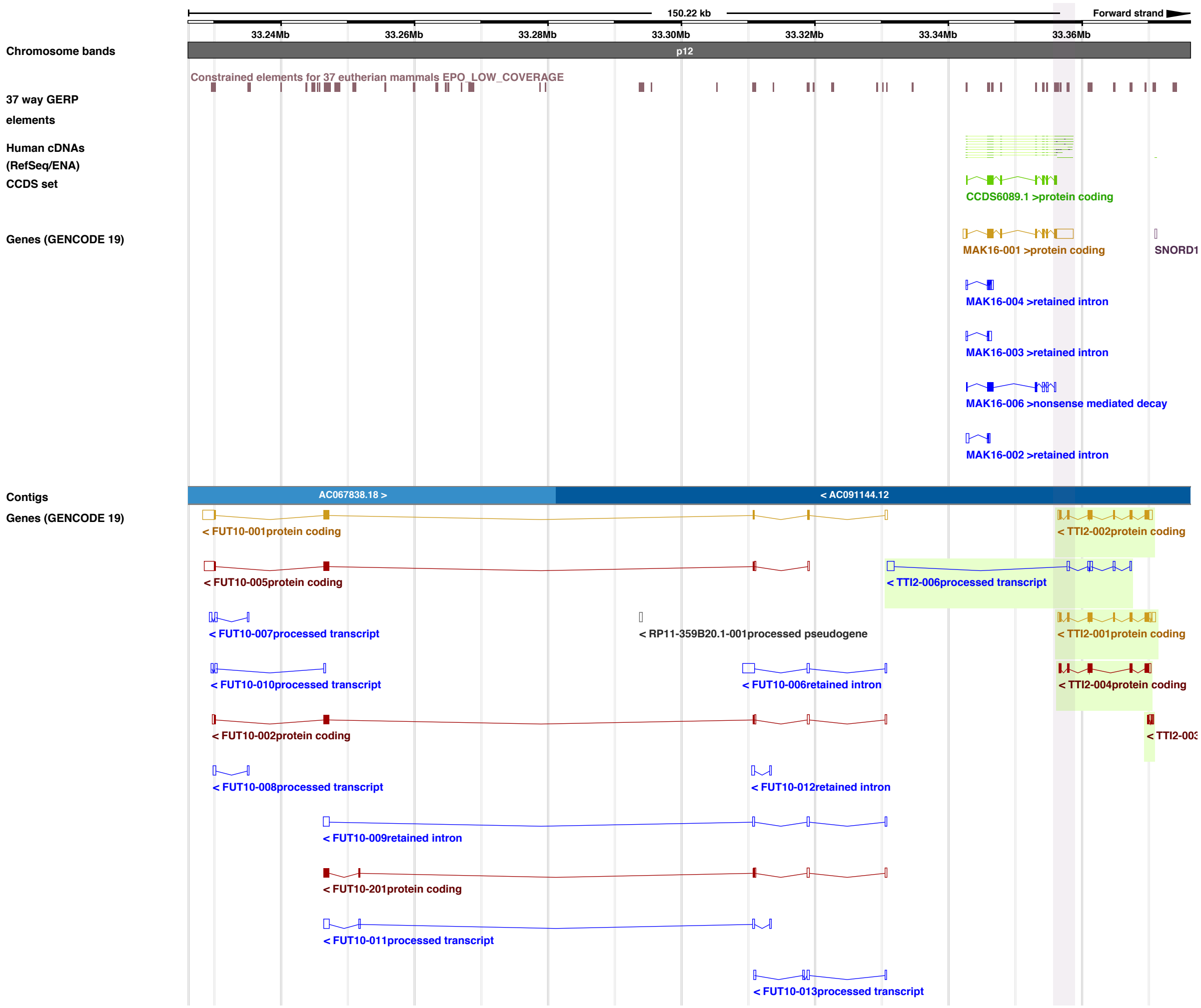

Supplementary Figure 6. Genetic region of chromosome 8 encompassing *FUT10*, *MAK16* and *TTI2* genes. The thick vertical light grey frame with coordinates 33,342,268-33,358,778 shows overlap between exon 10 of MAK16 and exons 6 and 7 of TTI2. Blue: processed transcript; green: CCDS set; dark brown: Ensemble protein coding; beige: merged Ensembl/Havana; black: pseudogene. This image was retrieved from Ensembl.

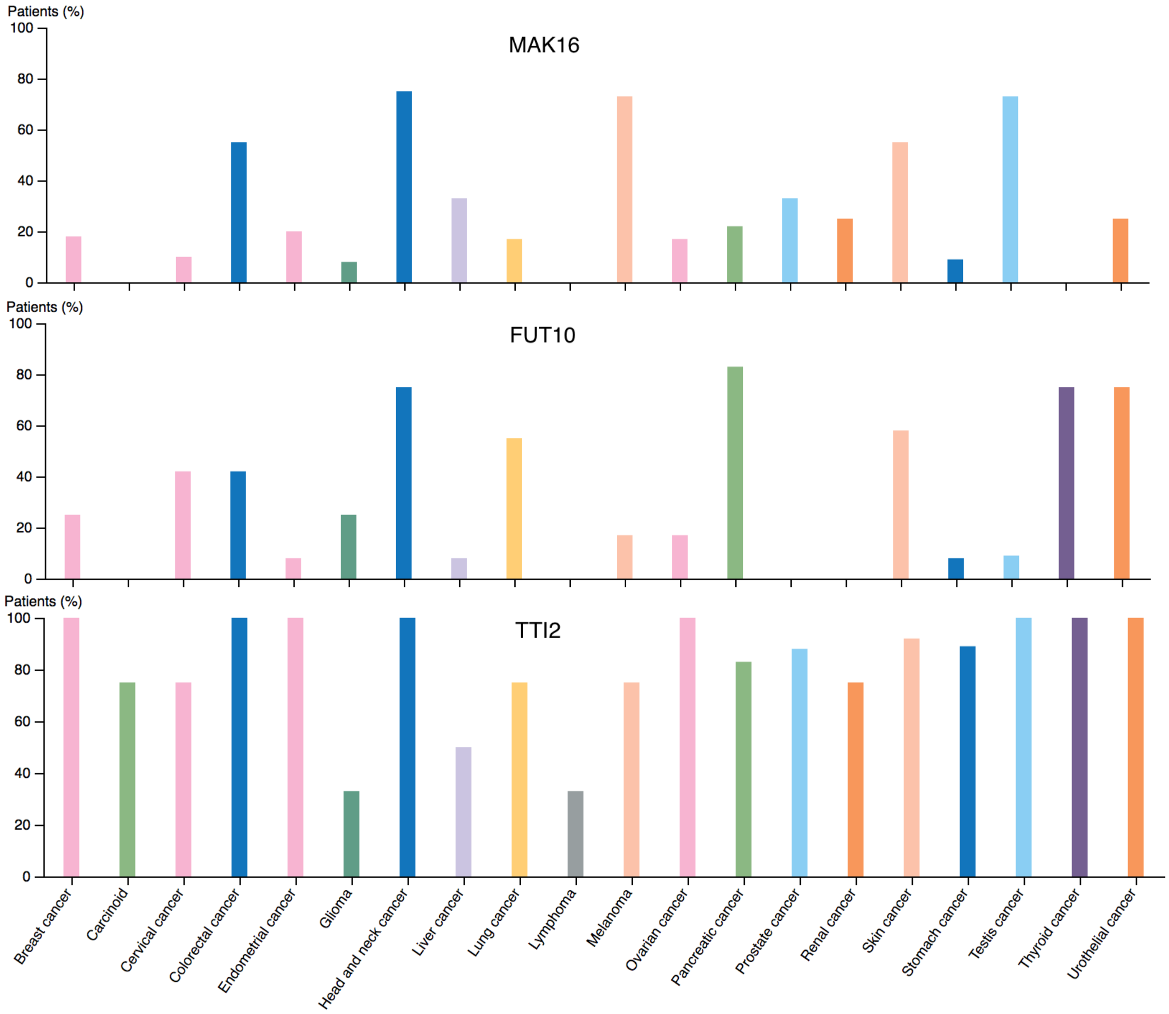

Supplementary Figure 7. Expression of proteins encoded by *MAK16*, *FUT10* and *TTI2* across several cancer types. Shown are the percentage of patients showing high/medium expression of these proteins. Expression was quantified by immunohistochemistry. A total of 12 patients were tested for immunoreactivity in urothelial cancer samples. These images were retrieved from the Human Protein Atlas webpage (`https://www.proteinatlas.org/`).

Supplementary Tables

Variables	Nº Cases (%)	Nº Controls (%)	*P*-value
Age, mean (SD)	66.13 (9.97)	65.21 (10.09)	0.53
Sex (%)			
Male	65 (0.71)	56 (0.6)	0.18
Female	27 (0.29)	37 (0.4)	
Total	92	93	
Occupational risk factors (%)			
No	63 (68.5)	76 (81.7)	0.055
Yes	29 (31.5)	17 (18.3)	
Mining workers (%)			
No	69 (75)	71 (76.3)	0.12
Yes	23 (25)	12 (23.7)	
Smoking status (%)			
Never Smoker	37 (40.2)	37 (39.8)	1
Ever Smoker	55 (59.8)	56 (60.2)	

Supplementary Table 1. Distribution of host characteristics according to case-control status. Shown are the distributions for age, sex, occupational risk factors for BC (ORFs) and smoking status. Because mining workers represent most ORFs, mining workers's status was also evaluated separately. Differences in age between cases and controls were evaluated using a two-tailed t-test, whereas for the remaining variables we used a chi-squared test of independece; DF = 1; $P < 0.05$ was considered significant.

Top $\mathbf{PBS}_{n1}$ loci	Mean ***P*-value** ***selected***	Mean ***P*-value** ***random***	***P*-value** ***t*-test**
Top 5	0.220	0.490	0.090
Top 10	0.269	0.493	0.022*
Top 25	0.419	0.495	0.143
Top 50	0.407	0.495	0.028*
Top 100	0.401	0.495	0.001*

Supplementary Table 2. Summary statistics of *t*-test comparing mean BC association *P*-values from $\mathbf{PBS}_{n1}$-selected and random set of loci. $^*P < 0.05$ was considered significant.

**Population**	**Group**	**n**	**Source**
Atacama	ATA	185	this study
Aymara	MA	25	Bigham et al Bigham:2010aa
Quechua	MA	24	Bigham et al Bigham:2010aa
Nahua, Mixtec, Tlapanec	MA	14	Bigham et al Bigham:2010aa
Maya	MA	25	Bigham et al Bigham:2010aa
North Europe	EUR	85	1000GP
Britain	EUR	89	1000GP
Iberians from Spain	EUR	14	1000GP
Spain	EUR	625	POPRES
Toscani in Italy	EUR	98	1000GP
African from US	AFR	56	1000GP
Luhya in Webuye, Kenya	AFR	85	1000GP
Yoruba in Ibadan, Nigeria	AFR	88	1000GP
Han Chinese in Beijing, China	EAS	97	1000GP
Japanese in Tokyo, Japan	EAS	98	1000GP

Supplementary Table 3. Summary of populations used in this study. MA, EAS, Spain and ATA groups were used for the PBS_{n1} test. MA, EUR, AFR and ATA groups were used for estimating global and local ancestry. n: sample size.

Supplementary File Legends

Supplementary File 1. Variants with enrichment in mean local Native-American ancestry. Variants with significant deviations in the mean local Native-American ancestry over the genome-wide mean in the whole cohort and in controls considered separately but not in cases considered separately. Shown are the corresponding SNP ID, physical position, chomosome, P-value of the t-test obtained using the whole cohort, associated gene, SO consequence type and biotype. Genes associated with intergenic variants are not shown. Abbreviations: MIS: missense; SYN: synonymous; DS: downstream; US: upstream; IN: intron; IN-NC: intron/non-coding transcript; IG: intergenic; NC: non-coding transcript; NCE-NC: non coding transcript exon/non coding transcript; IN-NMD: intron/nonsense mediated decay; REG: regulatory region; PC: protein coding; AS: antisense; NMD: nonsense mediated decay transcript; PT: processed transcript; PP: processed pseudogene; UP: unprocessed pseudogene; PFR: promoter-flanking region.
