# Supplementary material for: Adaptation to Extreme Environments in an Admixed Human Population from the Atacama Desert
Source: Genome Biol Evol. 2019 Aug 6;11(9):2468–79. doi: 10.1093/gbe/evz172 (PMC6733355; doi:10.1093/gbe/evz172)
Supplement: evz172_Supplementary_Data [file evz172_supplementary_data.zip › Supplementary_File_1.pdf]

SNP_ID	POSITION	CHR	P_VALUE	GENE	CONSEQ	BIOTYPE
rs6468171	33356074	8	1.31E-09	*MAK16*	MISS	PC
rs2676419	33355886	8	1.31E-09	*MAK16*	IN	PC
rs1829189	33350296	8	1.31E-09	*MAK16*	IN	PC
rs2676403	33348000	8	1.31E-09	*MAK16*	IN	PC
rs2732303	33303016	8	1.31E-09	*FUT10*	IN	PC
rs2468549	33301621	8	1.31E-09	*FUT10*	IN	PC
rs10464811	33300773	8	1.31E-09	*FUT10*	IN	PC
rs938641	33296969	8	1.31E-09	*FUT10*	IN	PC
rs2732256	33295699	8	1.31E-09	*FUT10*	IN	PC
rs6982159	33292469	8	1.31E-09	*FUT10*	IN	PC
rs2732259	33292032	8	1.31E-09	*FUT10*	IN	PC
rs12155581	33287802	8	1.31E-09	*FUT10*	IN	PC
rs2459516	33284007	8	1.31E-09	*FUT10*	IN	PC
rs9656765	33274426	8	1.31E-09	*FUT10*	IN	PC
rs16880933	33271336	8	1.31E-09	*FUT10*	IN	PC
rs7813318	33269745	8	1.31E-09	*FUT10*	IN	PC
rs12549316	33264711	8	1.31E-09	*FUT10*	IN	PC
rs6998432	33260717	8	1.31E-09	*FUT10*	IN	PC
rs7007756	33250176	8	1.31E-09	*FUT10*	IN	PC
rs7005921	33249796	8	1.31E-09	*FUT10*	IN	PC
rs16880852	33246685	8	1.31E-09	*FUT10*	SYN	PC
rs16880851	33246562	8	1.31E-09	*FUT10*	SYN	PC
rs16880846	33245799	8	1.31E-09	*FUT10*	IN	PC
rs16880842	33245636	8	1.31E-09	*FUT10*	IN	PC
rs1568117	33242939	8	1.31E-09	*FUT10*	IN	PC
rs7840631	33235362	8	1.31E-09	*FUT10*	IN	PC
rs7012873	33228199	8	1.31E-09	*FUT10*	DS	PC
rs1474035	33228162	8	1.31E-09	*FUT10*	DS	PC
rs6993806	33325171	8	1.31E-09	*FUT10*	IN	PC
rs2732315	33313329	8	1.31E-09	*FUT10*	IN	PC
rs6995454	33303493	8	1.31E-09	*FUT10*	IN	PC
rs905309	45161016	18	2.78E-08	*TPMTP1*	DS	PP
rs10502885	45155501	18	2.78E-08	*TPMTP1*	US	PP
rs984360	45155234	18	2.78E-08	*TPMTP1*	US	PP
rs426224	45154351	18	2.78E-08	*TPMTP1*	US	PP
rs984655	45151919	18	2.78E-08	*TPMTP1*	US	PP
rs1510019	45140729	18	2.78E-08		IG	-
rs16955746	45140104	18	2.78E-08		IG	-
rs9945915	45139487	18	2.78E-08		IG	-
rs6507779	45138254	18	2.78E-08		IG	-
rs12967226	45138216	18	2.78E-08		IG	-
rs7240934	45138173	18	2.78E-08		IG	-
rs11082611	45134328	18	2.78E-08		IG	-
rs2175222	45132520	18	2.78E-08		IG	-
rs3968537	45132081	18	2.78E-08		IG	-
rs11665292	45131800	18	2.78E-08		IG	-
rs9944793	45130611	18	2.78E-08		IG	-
rs11661022	45125322	18	2.78E-08	*CTD-2130O13.1*	DS	lincRNA
rs952233	45118805	18	2.78E-08	*CTD-2130O13.1*	DS	lincRNA

SNP_ID	POSITION	CHR	P_VALUE	GENE	CONSEQ	BIOTYPE
rs12327437	45117529	18	2.78E-08	*CTD-2130O13.1*	DS	lincRNA
rs11877554	45116902	18	2.78E-08	*CTD-2130O13.1*	DS	lincRNA
rs11661651	45110701	18	2.78E-08	*CTD-2130O13.1*	IN-NC	lincRNA
rs7802268	108674727	7	8.71E-08		IG	-
rs7796746	108673515	7	8.71E-08		IG	-
rs175691	108669555	7	8.71E-08		IG	-
rs10280128	108660736	7	8.71E-08		IG	-
rs275535	108660398	7	8.71E-08		IG	-
rs12534710	108647398	7	8.71E-08		IG	-
rs175693	108607049	7	8.71E-08		IG	-
rs41427546	108606684	7	8.71E-08		IG	-
rs1513921	108606430	7	8.71E-08		IG	-
rs11768328	108596014	7	8.71E-08	*AC004014.3*	DS	lincRNA
rs275551	108595645	7	8.71E-08	*AC004014.3*	DS	lincRNA
rs848342	108560313	7	8.71E-08	*AC004014.3*	IN-NC	lincRNA
rs1100210	108559304	7	8.71E-08	*AC004014.3*	IN-NC	lincRNA
rs11773556	108551518	7	8.71E-08	*AC004014.3*	IN-NC	lincRNA
rs848353	108548660	7	8.71E-08	*AC004014.3*	US	lincRNA
rs860229	108541193	7	8.71E-08	*FLJ00325*	US	PC
rs12705521	108534672	7	8.71E-08		IG	-
rs848374	108534194	7	8.71E-08		IG	-
rs1525205	108532718	7	8.71E-08		IG	-
rs9690118	108532180	7	8.71E-08		IG	-
rs848385	108531984	7	8.71E-08		IG	-
rs848386	108531829	7	8.71E-08		IG	-
rs12670622	108531733	7	8.71E-08		IG	-
rs3922355	108530220	7	8.71E-08		IG	-
rs848400	108525044	7	8.71E-08	*C7orf66*	US	PC
rs10278613	108524793	7	8.71E-08	*C7orf66*	US	PC
rs2280645	108524003	7	8.71E-08	*C7orf66*	DS	PC
rs848405	108521842	7	8.71E-08	*C7orf66*	DS	PC
rs848406	108520541	7	8.71E-08	*C7orf66*	DS	PC
rs711444	108510138	7	8.71E-08		IG	-
rs9649314	108506618	7	8.71E-08		IG	-
rs1525180	108505320	7	8.71E-08		IG	-
rs1525181	108505208	7	8.71E-08		IG	-
rs9690914	108497479	7	8.71E-08		IG	-
rs10275432	108483769	7	8.71E-08		IG	-
rs940719	108480705	7	8.71E-08		IG	-
rs1525186	108479722	7	8.71E-08		IG	-
rs1404692	108469460	7	8.71E-08		IG	-
rs10224425	108452177	7	8.71E-08		IG	-
rs17156209	108447883	7	8.71E-08		IG	-
rs10272265	108423245	7	8.71E-08		IG	-
rs16872610	108412614	7	8.71E-08		IG	-
rs10264277	108410815	7	8.71E-08		IG	-
rs3793324	108408695	7	8.71E-08		IG	-
rs9987062	108408111	7	8.71E-08		IG	-

SNP_ID	POSITION	CHR	P_VALUE	GENE	CONSEQ	BIOTYPE
rs17156165	108408007	7	8.71E-08		IG	-
rs7791522	108401495	7	8.71E-08		IG	-
rs10251803	108398777	7	8.71E-08		IG	-
rs7802393	108394903	7	8.71E-08		IG	-
rs6966292	108390955	7	8.71E-08		IG	-
rs6966215	108390750	7	8.71E-08		IG	-
rs6967009	108385515	7	8.71E-08		IG	-
rs12112172	108384471	7	8.71E-08		IG	-
rs6977586	108384142	7	8.71E-08		IG	-
rs6466250	108380331	7	8.71E-08		IG	-
rs2396018	108380208	7	8.71E-08		IG	-
rs16872605	108379752	7	8.71E-08		IG	-
rs2214129	108378117	7	8.71E-08		IG	-
rs7836942	33380685	8	8.71E-08	*RP11-359B20.5*	NCE-NC	PP
rs2732287	33376199	8	8.71E-08	*RP11-359B20.5*	US	PP
rs6989048	33369082	8	8.71E-08	*TTI2*	IN	PC
rs2188598	108377828	7	1.52E-07		IG	-
rs4730331	108377311	7	1.52E-07		IG	-
rs4730329	108377076	7	1.52E-07		IG	-
rs2852948	45245865	18	7.53E-07		IG	-
rs12969463	45244578	18	7.53E-07		IG	-
rs905311	45244296	18	7.53E-07		IG	-
rs905310	45243999	18	7.53E-07		IG	-
rs4510095	45239288	18	7.53E-07		IG	-
rs4939877	45238757	18	7.53E-07		IG	-
rs4939876	45238661	18	7.53E-07		IG	-
rs16957302	45237816	18	7.53E-07		IG	-
rs7233486	45228744	18	7.53E-07	*RP11-767C4.1*	US	PP
rs12604433	45227789	18	7.53E-07	*RP11-767C4.1*	US	PP
rs905313	45207893	18	7.53E-07		IG	-
rs16956699	45197730	18	7.53E-07		IG	-
rs16956696	45196373	18	7.53E-07		IG	-
rs9958500	45195937	18	7.53E-07		IG	-
rs9960268	45184546	18	7.53E-07		IG	-
rs4319835	45183961	18	7.53E-07		IG	-
rs17737898	45177508	18	7.53E-07		IG	-
rs1995415	45175402	18	7.53E-07		IG	-
rs10732353	31568897	9	5.48E-06		IG	-
rs10758076	31568379	9	5.48E-06		IG	-
rs16917154	31568046	9	5.48E-06		IG	-
rs10970421	31561341	9	5.48E-06		IG	-
rs10813617	31551036	9	5.48E-06		IG	-
rs10813616	31550664	9	5.48E-06		IG	-
rs10758070	31539194	9	5.48E-06		IG	-
rs4879523	31534673	9	5.48E-06		IG	-
rs2502176	31533362	9	5.48E-06		IG	-
rs2502177	31532933	9	5.48E-06		IG	-
rs974913	31527272	9	5.48E-06		IG	-

SNP_ID	POSITION	CHR	P_VALUE	GENE	CONSEQ	BIOTYPE
rs2481583	31525217	9	5.48E-06		IG	-
rs2502193	31523416	9	5.48E-06		IG	-
rs1014925	31501040	9	5.48E-06	*RP11-291J9.2*	US	UP
rs16917048	31493267	9	5.48E-06		IG	-
rs16917043	31492927	9	5.48E-06		IG	-
rs16917039	31490611	9	5.48E-06		IG	-
rs10970383	31490538	9	5.48E-06		IG	-
rs1904131	31481298	9	5.48E-06		IG	-
rs7041035	31472315	9	5.48E-06		IG	-
rs10970375	31470558	9	5.48E-06		IG	-
rs10970374	31470393	9	5.48E-06		IG	-
rs10970373	31470293	9	5.48E-06		IG	-
rs11515349	31468724	9	5.48E-06		IG	-
rs7861554	31456573	9	5.48E-06		IG	-
rs10970345	31450384	9	5.48E-06		IG	-
rs7864619	31448964	9	5.48E-06		IG	-
rs7856016	31446664	9	5.48E-06		IG	-
rs10813582	31446639	9	5.48E-06		IG	-
rs10813581	31446175	9	5.48E-06		IG	-
rs10118531	31434342	9	5.48E-06		IG	-
rs6476287	31418064	9	5.48E-06		IG	-
rs10813575	31411344	9	5.48E-06		IG	-
rs12235995	31399459	9	5.48E-06		IG	-
rs7870771	31397582	9	5.48E-06		IG	-
rs10758058	31396656	9	5.48E-06		IG	-
rs12379656	31383712	9	5.48E-06	*RP11-271O3.1*	US	lincRNA
rs1481208	31381642	9	5.48E-06	*RP11-271O3.1*	US	lincRNA
rs1481207	31381459	9	5.48E-06	*RP11-271O3.1*	NCE-NC	lincRNA
rs7855520	31381316	9	5.48E-06	*RP11-271O3.1*	IN-NC	lincRNA
rs10970317	31381021	9	5.48E-06	*RP11-271O3.1*	IN-NC	lincRNA
rs964929	31364336	9	5.48E-06		IG	-
rs12350816	31354658	9	5.48E-06		IG	-
rs6476284	31347460	9	5.48E-06		REG	PFR
rs12006512	31335967	9	5.48E-06		IG	-
rs10813538	31335888	9	5.48E-06		IG	-
rs10120907	31324281	9	5.48E-06		IG	-
rs7044054	31321155	9	5.48E-06		IG	-
rs10970259	31290253	9	5.48E-06		IG	-
rs12344352	31270964	9	5.48E-06		IG	-
rs957594	31356441	9	5.48E-06		IG	-
rs10970260	31291138	9	5.48E-06		IG	-
rs7873731	31586339	9	5.48E-06		IG	-
rs10813623	31577568	9	5.48E-06		IG	-
rs4879525	31574888	9	5.48E-06		IG	-
